# Therapeutic synergy in long-standing plasma cell gingivitis: integrating photobiomodulation and immunomodulatory management^؆^

**DOI:** 10.1016/j.abd.2026.501354

**Published:** 2026-05-13

**Authors:** Josefina Hurtado, Víctor Meza, Fernando Valenzuela, Isidora Mujica

**Affiliations:** a Department of Oral Pathology, Faculty of Dentistry, Universidad de los Andes, Santiago, Chile; b Department of Dermatology, Faculty of Medicine, Universidad de los Andes, Santiago, Chile; c Department of Dermatology, Clínica Universidad de los Andes, Santiago, Chile; d Department of Dermatology, Faculty of Medicine, University of Chile, Santiago, Chile

*Dear Editor,*

Plasma Cell Gingivitis (PCG) is a rare chronic inflammatory gingival disorder characterized by a dense subepithelial infiltrate of mature plasma cells and marked erythema, unrelated to biofilm accumulation. Its multifactorial etiology includes hypersensitivity reactions, systemic conditions, and immune dysregulation.^1^ Recent evidence suggests that it may mimic or overlap with Autoimmune Mucocutaneous Disorders (AMDs) (including oral lichen planus and mucous membrane pemphigoid), which can manifest as desquamative gingivitis with plasma-cell-rich infiltrates, highlighting the need for histopathologic analysis before confirming PCG.^2^

A 39-year-old woman with a five-year history of persistent gingival inflammation (Fig. 1) unresponsive to conventional periodontal therapy, was referred to the Oral Pathology service for further assessment. Her medical history included untreated hypercholesterolemia and hypothyroidism, dysautonomia, keloid formation, and a family history of rheumatoid arthritis. At initial examination, the gingival tissues were free of plaque and calculus, excluding plaque-induced periodontal disease. The working diagnosis initially considered AMDs; however, the bright scarlet erythema with a velvety surface, in the absence of Wickham striae or vesiculobullous lesions, strongly suggested PCG. Extensive blood testing excluded autoimmune diseases. The patient’s untreated conditions were recognized as potential contributors to immune dysregulation. To address this, management with rosuvastatin (10 mg/day) and levothyroxine (25 µg/day) was initiated, aiming to restore metabolic balance and reduce systemic inflammation.

**Fig. 1:**
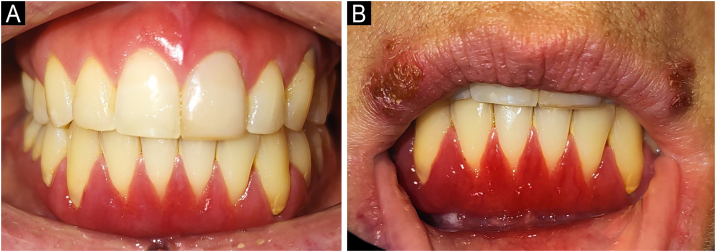
Clinical presentation and early treatment phase of PGC. (A) Baseline presentation showing chronic inflammation of marginal and attached gingiva with pronounced erythema, edema, loss of knife-edge contour, and superficial erosion. (B) Bilateral herpes labialis in crusting stage, three weeks after initiation of topical tacrolimus 0.1% mouthwash. Gingival erythema remains unchanged at this stage; PBMT was subsequently introduced as adjunctive therapy.

An incisional punch biopsy was performed. Histopathologic examination revealed stratified squamous epithelium with focal erosion and spongiosis, accompanied by a dense subepithelial infiltrate of mature plasma cells within the lamina propria. Immunohistochemical staining demonstrated a polyclonal pattern, with positive reactivity for both kappa and lambda light chains, thereby excluding plasma cell neoplasia and autoimmune diseases, and confirming the diagnosis of PCG (Fig. 2).^3^

**Fig. 2:**
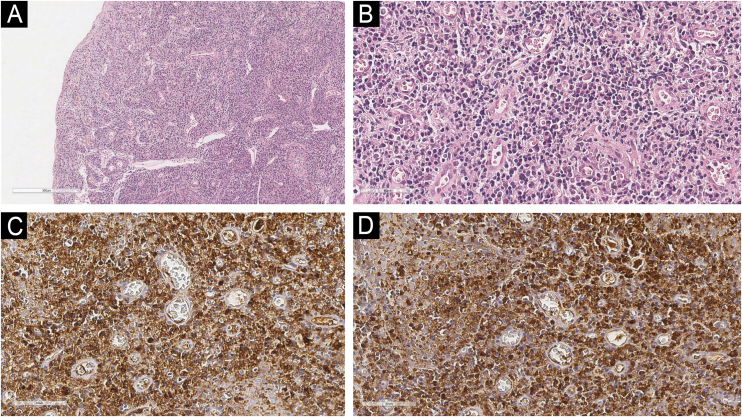
Histopathological and immunohistochemical findings. (A) Stratified squamous epithelium with surface erosion and spongiosis (Hematoxilyn & eosin, 100×). (B) Dense subepithelial plasma-cell infiltrate with eccentric nuclei and basophilic cytoplasm (Hematoxilyn & eosin, 400×). (C) Positive cytoplasmic staining for kappa light chains in plasma cells (immunostaining, digital magnification equivalent to 400×). (D) Positive cytoplasmic staining for lambda light chains confirming polyclonality (immunostaining, digital magnification equivalent to 400×).

Further dermatological evaluation ruled out mucocutaneous or gastrointestinal involvement. Patch testing revealed hypersensitivity to cinnamon, honey, wheat flour, nickel, methylisothiazolinone, and thimerosal. Allergen elimination was implemented as the primary therapeutic measure,^1^ and topical tacrolimus 0.1% mouthwash was prescribed twice daily for one month as an initial immunomodulatory approach, given its efficacy in plasma cell-mediated mucosal inflammation.^4^ Additionally, nutritional support with vitamin C, vitamin E, and omega-3 fatty acids was prescribed to promote mucosal healing.^5^

However, prolonged tacrolimus use was limited by its high cost, risk of gingival atrophy, and herpes labialis reactivation in the third week (Fig. 1B).^6^ To reduce drug exposure while maintaining inflammation control, Photobiomodulation Therapy (PBMT) was added three times weekly for 8-weeks using a portable dual-wavelength diode laser (LASER DUO, MM Optics Ltda., São Carlos, Brazil) (LASER DUO, MM Optics, São Carlos, Brazil; 660 nm InGaAlP, continuous mode). Each site received 4 J at 100 mW for 40 s (133 J/cm^2^). All irradiation parameters are detailed in Supplementary Tables S1‒3.^7^

PBMT exerts its effects through multiple biological mechanisms. Photon absorption by cytochrome c oxidase enhances mitochondrial ATP synthesis, restoring cellular energy metabolism and promoting tissue repair. It transiently increases Reactive Oxygen Species (ROS), triggering adaptive antioxidant responses and redox-sensitive signaling. PBMT downregulates proinflammatory mediators such as IL-1β, IL-6, TNF-α, and PGE, while upregulating IL-10 and TGF-β. It also suppresses NF-κB activation, down-regulating the transcription of pro-inflammatory genes. Additional effects include vasodilation, angiogenesis, improved lymphatic drainage, and analgesia through modulation of nerve conduction and endorphin release. The proliferative effects on fibroblasts and keratinocytes accelerate epithelial healing, which is particularly beneficial in erosive lesions.^5^, ^8^, ^9^

PBMT produced a marked reduction in erythema, bleeding, and pain after the third session, with progressive normalization of gingival contour (Fig. 3). Its combination with short-term topical immunosuppression created a favorable environment for tissue repair, while allergen elimination addressed the underlying trigger. Correction of systemic comorbidities likely improved immune homeostasis. Notably, hypothyroidism reduces LDL receptor expression and promotes lipid oxidation, sustaining the proinflammatory state underlying PCG chronicity.^10^ Finally, a strict oral hygiene protocol was maintained, emphasizing professional plaque control and patient education, both essential for long-term stability. During the three-month follow-up, the patient exhibited complete clinical remission (Fig. 3). Regular follow-up by dermatology, endocrinology, and immunology services was maintained to prevent recurrence and to monitor systemic conditions.

**Fig. 3:**
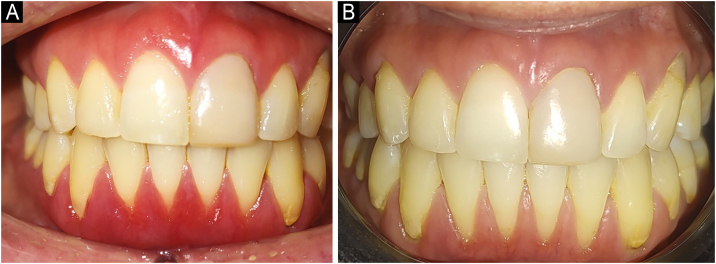
Clinical evolution: baseline and three-month post-treatment comparison. (A) Baseline clinical presentation (as shown in Fig. 1A). (B) Three-month follow-up showing resolution of erythema and edema, re-establishment of knife-edge contour, and residual recession due to previous gingival attachment loss.

This case illustrates that effective PCG management requires a multimodal approach. The integration of PMBT as an adjuvant therapy offers a non-invasive and well-tolerated option for managing the chronic inflammatory environment in PCG, minimizing prolonged tacrolimus or corticosteroid exposure and associated risks.^3^

## Authors' contributions

Josefina Hurtado: Literature search; draft writing and review.

Víctor Meza: Review and editing, and visualization.

Fernando Valenzuela: Project conception; draft writing, review and editing, and visualization.

Isidora Mujica: Project conception; draft writing, review and editing, and visualization.

## Declaration of Generative AI and AI-assisted technologies in the writing process

During the preparation of this work, the authors used CLAUDE (Sonnet 4.5) in order to improve readability. After using this tool, the authors reviewed and edited the content as needed and take full responsibility for the content of the publication.

## Financial support

None declared.

## Research data availability

Does not apply.

## Conflicts of interest

None declared.
