# Supplementary material for: Therapeutic synergy in long-standing plasma cell gingivitis: integrating photobiomodulation and immunomodulatory management
Source: An Bras Dermatol. 2026 May 13;101(3):501354. doi: 10.1016/j.abd.2026.501354 (PMC13195699; doi:10.1016/j.abd.2026.501354)
Supplement: Supplementary file 1 [file mmc1.docx]

**ABD-D-25-00728**

**Supplementary Material**

**Table S1** Device information.

| **Manufacturer** | MMOptics Ltda. (São Carlos, São Paulo, Brazil) |
| --- | --- |
| Model Identifier | LASER Duo |
| Number of Emitters | Two (dual laser system) |
| Emitter Type | GaAlAs (Gallium–Aluminum–Arsenide) (808 nm, infrared) and InGaAlP (Indium–Gallium–Aluminum–Phosphide) (660 nm, red) semiconductor diode lasers |
| Spatial Distribution of Emitters | Two co-axial emitters integrated within a single handheld probe (one visible red, one infrared) |
| Beam Delivery System | Direct contact through interchangeable autoclavable tip (3 mm^2^ beam area) |

**Table S2** Irradiation parameters.

| **Parameter** | **Value** | **Measurement method or information source** |
| --- | --- | --- |
| Center wavelength [nm] | 660±10 nm (red, InGaAlP) | Manufacturer specification |
| Spectral bandwidth [nm] | ±10 nm | Manufacturer specification |
| Operating mode | Continuous wave (CW^a^) | Manual and device display |
| Frequency [Hz] | Not applicable (continuous mode) | ‒ |
| Pulse on duration [sec] | Not applicable (continuous wave) | ‒ |
| Pulse off duration [sec] or duty cycle [%] | Not applicable (continuous wave) | ‒ |
| Energy per pulse [J] | Not applicable (continuous wave) | ‒ |
| Peak radiant power [mW] | 100 mW ± 20% | Manufacturer specification |
| Average radiant power [mW] | 100 mW ± 20% | Manufacturer specification |
| Polarization | Linear | Manufacturer specification |
| Aperture diameter [cm] | 0.2 cm (3 mm^2^ area) | Manufacturer specification |
| Irradiance at aperture [mW/cm^2^] | ≈3.3 × 10^3^mW/cm^2^ | Calculated (Power/Area) |
| Beam divergence [rad or deg]^b^ | ‒ | Not specified by manufacturer |
| Beam shape^c^ | Circular | Manufacturer specification |
| Beam profile^d^ | Gaussian | Manufacturer specification |

^a^ CW (continuous wave) emission delivers a constant photon flux over time; therefore, pulsed parameters (pulse duration, off duration, energy per pulse) are not applicable.

^b^ Beam divergence not provided by manufacturer.

^c^ Beam cross-section is circular at the aperture.

^d^ Beam profile follows a Gaussian distribution.

**Table S3** Treatment parameters.

| **Parameter** | **Value** | **Additional notes** |
| --- | --- | --- |
| Beam spot size at target [cm^2^] | 0.03 cm^2^ | Direct contact tip, 3 mm^2^ beam area |
| Irradiance at target [mW/cm^2^] | 3.3 × 10^3^ mW/cm^2^ | Calculated from 100 mW / 0.03 cm^2^ |
| Exposure duration [sec] | 40s | Per irradiation point |
| Radiant exposure [J/cm^2^] | 133 J/cm^2^ | Calculated as (Power × time / area) = (0.1W × 40s / 0.03 cm^2^) |
| Radiant energy [J] | 4 J | Delivered per point |
| Number of points irradiated | 6-points | Distributed along affected gingival area (3-points in maxillary gingiva and 3 in mandibular gingiva) |
| Area irradiated [cm^2^] | 0.18 cm^2^ | Six points, 0.03 cm^2^ each |
| Application technique | Contact with slight pressure, perpendicular to tissue | Hand-held probe, stationary |
| Number and frequency of treatment sessions | 24 sessions in total (three times per week for eight weeks) | As described in the protocol by Pires et al. [7] |
| Total radiant energy [J] | 576 J | 4 J × 6-points × 24-sessions |

All laser parameters refer to the 660 nm InGaAlP diode emitter (LASER DUO, MM Optics Ltda., São Carlos, São Paulo, Brazil). Radiant exposure (J/cm^2^) and total radiant energy (J) were calculated from the output power, exposure time, and beam spot size. The treatment protocol was adapted from Pires et al.,[7] preserving similar irradiance and fluence parameters. Modifications included the number of irradiation points (six gingival sites), the frequency and total number of sessions (three times per week for eight weeks, totaling 24-sessions), and the total irradiated area, which was adjusted to match the clinical extent of the gingival lesion. The 3 mm^2^ beam area (0.03 cm^2^) corresponds to an approximate beam diameter of 1.95 mm. Considering the ±20% output power uncertainty reported by the manufacturer, the actual radiant exposure may range between approximately 106‒160 J/cm^2^, although this variability does not affect the internal consistency of the reported calculations. All parameters refer exclusively to the 660 nm InGaAlP diode; the 808 nm emitter remained inactive during treatment.

**Supplementary Data ‒ Digital Histopathology Slides.**

The following Aperio-scanned slides are available for open access visualization:

| **Specimen/Stain** | **Magnification** | **Link (Data Repository)** |
| --- | --- | --- |
| Hematoxylin & Eosin (H&E) | 40× | https://pathpresenter.net/public/display?token=b0ebc86d |
| Immunohistochemistry – Kappa light chain | 40× | https://pathpresenter.net/public/display?token=edaad208 |
| Immunohistochemistry – Lambda light chain | 40× | https://pathpresenter.net/public/display?token=ef31c993 |

Slides were digitized using Aperio (Leica Biosystems) at 40× magnification. Access is provided for research and review purposes.
